# Personal vision: enhancing work engagement and the retention of women in the engineering profession

**DOI:** 10.3389/fpsyg.2014.01400

**Published:** 2014-12-08

**Authors:** Kathleen R. Buse, Diana Bilimoria

**Affiliations:** Weatherhead School of Management, Case Western Reserve UniversityCleveland, OH, USA

**Keywords:** personal vision, ideal self, engagement, self-efficacy, women engineers

## Abstract

This study examines how personal vision enhances work engagement and the retention of women in the engineering profession. Using a mixed method approach to understand the factors related to the retention of women in the engineering profession, we first interviewed women who persisted and women who opted out of the profession (Buse and Bilimoria, 2014). In these rich stories, we found that women who persisted had a personal vision that included their profession, and that this personal vision enabled them to overcome the bias, barriers and discrimination in the engineering workplace. To validate this finding on a larger population, we developed a scale to measure one's personal vision conceptualized as the ideal self (Boyatzis and Akrivou, 2006). The measure was tested in a pilot study and then used in a study of 495 women with engineering degrees. The findings validate that the ideal self is comprised of self-efficacy, hope, optimism and core identity. For these women, the ideal self directly impacts work engagement and work engagement directly impacts career commitment to engineering. The findings add to extant theory related to the role of personal vision and intentional change theory. From a practical perspective, these findings will aid efforts to retain women in engineering and other STEM professions.

## Introduction

“We've always been reluctant to publish numbers about the diversity of our workforce at Google. We now realize we were wrong, and that it's time to be candid about the issues. Put simply, Google is not where we want to be when it comes to diversity, and it's hard to address these kinds of challenges if you're not prepared to discuss them openly, and with the facts. So, here are our numbers… ”

With this statement posted on its website in May 2014, Google admitted that its international workforce was 70% men and in the US, 61% white (Google, 2014). Beyond that, Google committed to increasing diversity and relevant to our study, discussed its commitment to retaining and advancing women. This announcement set off an avalanche in the technology world with at least 13 other technology corporations voluntarily sharing their diversity statistics and also committing to create a more inclusive workplace (Mangalindan, 2014).

At a time when the US labor force is 47% women and 52% of managers and professionals are women (Bureau of Labor Statistics, 2014a), we seek to understand those factors that enable women to achieve in technology-driven organizations where women continue to be under-represented. We focus on women in engineering because it is the profession where women are most under-represented—only 10% in 2013, according to the US Bureau of Labor Statistics.

Researchers describe the under-representation of women in science, technology, engineering and math (STEM) as complex and the result of the interplay between individual, institutional, social and cultural factors (National Research Council, 2007). However, the few studies available on professional STEM women working in industry focus on why women leave (Frehill, 2008; Hewlett et al., 2008; Singh et al., 2013).

Using a mixed method approach, this study seeks to understand how and when women persist in STEM careers. We begin by using narratives from a qualitative study on women who persisted and women who left the engineering profession (Buse and Bilimoria, 2014). We use these narratives to frame a model built on theories of the ideal self as a personal vision (Boyatzis and Akrivou, 2006), engagement (Kahn, 1990), and the kaleidoscope career (Mainiero and Sullivan, 2005). We empirically test this model with a sample of 495 women with engineering degrees. We argue that understanding those women who do persist will not only aid in developing practical interventions to support the retention of women in engineering and other STEM professions, but also adds to theory development related to personal visioning, engagement, and women's careers.

## Theory, hypotheses, and methods

### Theory development and hypotheses

More than 80% of all engineers are employed in business and industry (National Science Foundation, 2011). Many of the firms that employ women as engineers also employ women in other professions where women are well represented including accountants and auditors (60%), human resources (70%), public relations managers (60%) and purchasing agents (67%). And while women leave these professions (Hewlett and Luce, 2005), the rate of exodus is higher in the STEM professions (Hewlett et al., 2008). The available studies on women engineers describe a difficult work environment (Jorgenson, 2002; Miller, 2004; Gill et al., 2008; Powers et al., 2009; Watts, 2009). Bias, barriers and discrimination confront women in the workplace, resulting in a decision to leave the engineering profession (Frehill, 2008; Hewlett et al., 2008; Singh et al., 2013).

**Figure 1:**
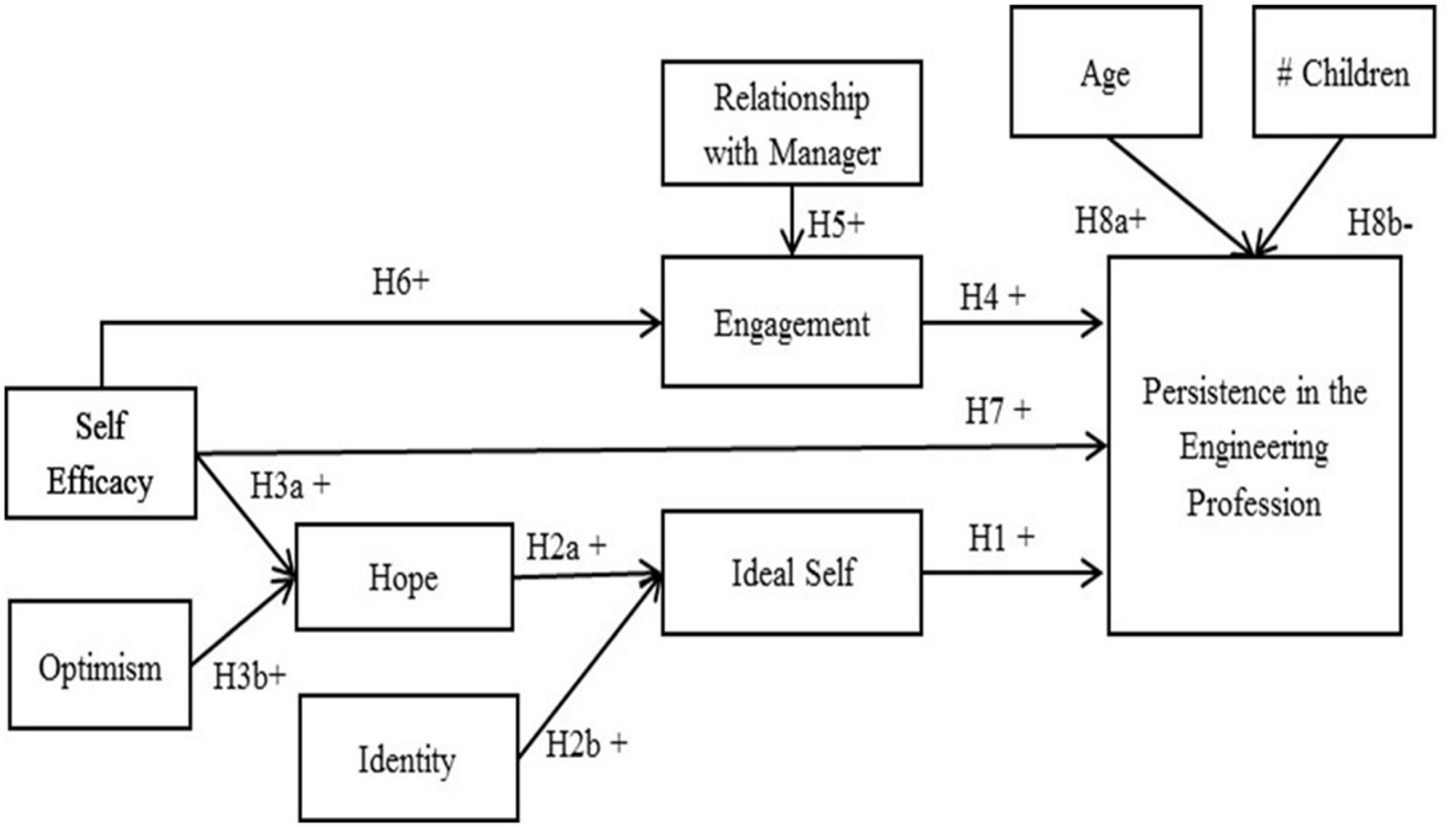
**Hypothesized model of factors impacting women's persistence in engineering**.

Despite the well documented difficulties in the workplace, some women do persist in these technology-driven and male-dominated workplaces. Because of the lack of theoretical frameworks related to career persistence, especially for women professionals, this work began with a qualitative research study including semi-structured interviews with women in the engineering profession: 21 women who persisted and 10 who opted out (see Buse et al., 2013). From a detailed analysis of the narratives, we provide evidence to develop the hypothesized model (Figure 1) that explains persistence for women in the engineering profession, taking as given the generally difficult work environments faced by women engineers as described above and extensively in the literature.

### The ideal self as personal vision

In this section, we begin by sharing examples of the narratives of the women engineers discussing their career decisions. We next discuss the intentional change theory, or ICT (Boyatzis, 2008), and show how these narratives can be explained using the ICT and in particular, how one's personal vision conceptualized as the ideal self-impacts career decisions for women engineers. This section ends with three hypotheses on factors impacting the ideal self, and how the ideal self-impacts career persistence.

A chemical engineer with more than 17 years' experience left the profession and went back to school to be certified as a math teacher. When we interviewed her, she was teaching 6th grade math in the school her three sons attended. Here, she discusses how her ideal self did not include her profession as an engineer. The sudden passing of her father caused deep reflection, motivating her to leave the engineering profession.

*There are certain things that kind of trigger (change) in life; I think you reflect upon really doing what you should be doing, and my dad passed away suddenly in May of 2005. What am I doing with my kids? … what do I want to be, and I really didn't feel like I was giving back enough because so much of your time is spent at work and not really concerned in serving the community…. I realized I wasn't happy anymore*.

Here, a former engineer, now a college business professor, describes how her work as an engineer did not align with her personal vison of leaving a legacy and impacting others. She describes a stark tipping point allowing her to realize this lack of alignment and motivating her to leave engineering.

*And he holds it up, and there's a list of maybe 15 names, each one has a date next to it that goes back at least one name per year on this list—maybe two names in one year or something. And I said, “What's this a list of?” And he goes, “This is the list of the suicides in this company.” … I wanted to leave a legacy, and all I saw was stock prices going up and down, but because I was so detached from how what I did was affecting the world in any way, it just—that's why my soul hurt. (Now) I've got this ability to make an impact (as a college business professor)*.

And, a self-described stay-at-home mom with 11 years in engineering describes how her personal vision did not include her engineering career once she became a mother. The birth of her first child was the tipping point for leaving engineering.

*It (engineering) really wasn't what I wanted to do. It was just something to get out of mom's house… I always knew I would leave. Because my mom worked, so that was something I'd always planned to stay home with my child*.

Intentional change theory (ICT), as theorized by Boyatzis (2008), includes the ideal self and the real self as two discoveries important to an individual's efforts to intentionally develop skills and to sustain any desired change. First, conceptualized as the basis for internal, emotional comparisons (Kolb and Boyatzis, 1970; Higgins, 1987), the ideal self can be described as one's personal vision, specifically about who the person wants to be and what she would like to accomplish in life. The real self is who the person is at the present time. When one recognizes discrepancies between the ideal self and the real self, a motivational force for change occurs. Often, these discrepancies are described as tipping points (Gladwell, 2000).

In our interviews with women who opted out of the engineering profession, we found support for this theory of change. Conversely, when the ideal self and the real self are in-sync, there is motivation for maintaining a current state. Those women who persisted in engineering, described themselves in engineering terms, as expressed by an enginering consultant with 28 years in the profession:

*I'm a hopeless geek. I can really—I love solving problems. I love working with users. My husband tells me that it's like I am so analytical about everything that he just wants to run from the room screaming sometimes. I love to solve problems; I actually have the toolset now, where the technology generally is easy for me. And I'm actually really, really good with people and facilitating communications among disparate groups*.

And, an engineering manager with 28 years of experience discussed her work:

*I really like what I do. There was just something about these industrial gas plants I just really like and enjoy. I don't know how to explain it. I just have a passion for the plants*.

According to the intentional change theory (ICT), the person engages in behavior in pursuit of their desired end state or the ideal self (Boyatzis, 2008). Efforts and, at times, sacrifices are made in the short term to accomplish more important longer-term goals as reflected in the ideal self (Boyatzis and Akrivou, 2006). In this study, it is proposed that the ability to maintain a current state or career requires an investment of energy toward a personal vision of oneself in that profession. This aspect of the ideal self has the potential to explain why women persist in a male-dominated profession, especially in light of the literature describing the extreme difficulties women experience (Frehill, 2008; Hewlett et al., 2008; Fouad and Singh, 2011). Thus, we hypothesize that:

***Hypothesis 1: The ideal self positively impacts persistence in the engineering profession***.

The ideal self is considered to have three major components: an image of a desired future, hope, and one's core identity (Boyatzis, 2008). This study focuses on hope and identity. Hope is defined as the feeling that something desirable is likely to happen and is proposed by Boyatzis as constituted by self-efficacy and optimism. In other words, hope is the perceived capability to derive pathways to desired goals and motivate oneself to those pathways (Snyder et al., 1996). As a result, we hypothesize:

***Hypothesis 2a: Hope positively impacts the ideal self***.

The concept of the core identity comes from strength-based approaches and is the awareness of one's strengths. Identity is defined as an unconscious set of enduring individual characteristics and includes one's strengths, context and resources. Identity is relatively stable and is a compilation of a person's enduring dispositions, involving a set of individual characteristics. Core identity is theorized as the third component of the ideal self and is defined as one's set of enduring individual characteristics (Boyatzis and Akrivou, 2006). The core identity is theorized to be relatively stable over time and includes one's roles, underlying the historical and continuing aspects of a person's ideal self. As a result, we hypothesize:

***Hypothesis 2b: Identity positively impacts the ideal self***.

Higher levels of individual resourcefulness lead to congruence between goals and achievements (Bakker and Demerouti, 2008). Self-efficacy and optimism have been acknowledged as some of these personal resources. Hope relates to goals and the identification of strategies to achieve these goals (Gallagher and Lopez, 2009). The ideal self is hypothesized as being emotionally powered by hope, where hope is caused by one's level of self-efficacy and optimism (Boyatzis and Akrivou, 2006). Thus, we hypothesize that:

***Hypothesis 3a: Self-efficacy positively impacts hope***.

***Hypothesis 3b: Optimism positively impacts hope***.

### Engagement

The question, “Why have you stayed in your engineering career?,” provided detailed descriptions of meaningful and challenging work that resulted in making the women engineers feel valuable because of the unique skills they brought to the workplace. Many provided examples of situations where they influenced a course of events and where their interactions with others provided a sense of accomplishment. These accounts fit Kahn's theory of work engagement (Kahn, 1990) and we build three hypotheses related to engagement, as shown in in Figure 1.

An engineering consultant who worked 28 years in the engineering profession told us she persisted because she found meaning and purpose in her work developing technology for military applications related to communications and intelligence:

*I can keep doing this. I can put up with all the crap. And then it sort of hit me that everybody is somebody's brother or father or son or cousin or whatever. And every single one of these guys and gals is there. Whatever it is that I can help bring to the fight to make sure that they're coming home to their three little toddlers or whatever*.

A process engineer with 16 years of engineering experience discussed feeling valuable and the challenges of her work as a process engineer in a manufacturing facility:

*I feel needed. I feel like if I didn't show up to work—maybe not one day; maybe not a week, but if I was gone for a month, I would be missed. There are a lot of things that I can do, that I'm the only person who can do those things. The controls, I did the programming (to control a new machine) all on my own, I was really proud of that… I stay in engineering because I just really love it. I love it! I love the challenge. I love coming in and having different things to do every single day. I love the people that I work with; I have a great support team*.

An RandD manager with 18 years of experience discussed the challenges, the novelty, and fun in engineering:

*I enjoy what I do. I enjoy the challenges. I enjoy the people. I like the fact I can travel and see something new… So I couldn't imagine doing something else. I can't imagine anything else that would be this much fun on a regular basis*.

A manufacturing manager with 25 years of experience discussed a temporary leave from her engineering career when she moved to France with her two children and her husband as he worked there on a 2-year assignment. Here, she describes why she returned to the engineering profession:

I really enjoy technical things. With two kids in college I need to be working and I want to be working. One of the things I really felt when I wasn't working after France was I felt like there was a part of me that was dying. I like technical challenges. I like thinking about things. I like working in spreadsheets and dealing with technical issues. That part of me wasn't being tapped into at all, and I really missed it. I did enjoy some of the other things I was doing, but I think my bigger passion is for technical things.

A process engineer, who was about to retire after a 30 year career in engineering, on why she stayed:

*Well, I think because the good times have definitely outweighed the bad ones, and it really is a very good group of people. I think having had the opportunity to do a number of different things keeps you from getting bored with it because just having the growth, being able to come in every day, and I'd learn something every day. Sometimes I'd learn a lot; sometimes I'd learn a little; sometimes I'd learn things I really didn't want to know. But at the end of the day, there's something new that you've taken back*.

Kahn (1990), credited with first defining the term engagement in a work role, theorized that work could provide a sense of meaning when employees felt worthwhile, useful and valuable. The meaningfulness is influenced by tasks, roles and work interactions. Tasks within a role that provide challenges or autonomy and have clear goals, influence engagement as do roles that carry status or influence. Work interactions that provide employees respect and a sense of worthwhileness promote engagement.

The notion of engagement in a work role has become popular in practice as engagement has been related to organizational benefits. Within the academic literature, the discussion of engagement is relatively new and is somewhat confusing as researchers provide various definitions and precede engagement with one of three modifiers: employee, job, or work. For example, Saks (2006, p. 603) uses the term employee engagement and defines it as a “distinct and unique construct that consists of cognitive, emotional and behavioral components that are associated with individual role performance.” Rich et al. (2010) use a similar definition, but use the term job engagement. Schaufeli and Bakker (2004) discuss engagement as the opposite of job burnout where engagement is characterized by vigor, dedication and absorption. High levels of energy, as well as a willingness to invest effort in one's work and to persist in the face of difficulties, characterize vigor. Where dedication is described as a sense of significance and challenge, absorption is characterized by being fully and happily focused in one's work. Sonnentag et al. (2012) prefer work engagement and define it as a positive and fulfilling work-related state of mind that is characterized by vigor, dedication and absorption. Others simply use the term “engagement” (Maslach and Leiter, 2008), as will be used here. No matter which term is used, engagement is portrayed as positive and fulfilling as related to a work role.

As Kahn (1990) described, engagement is an investment of cognitive, emotional and physical energies in role performance. It is a key mechanism that explains relationships between individual factors and benefits to organizations (Rich et al., 2010). Engagement is explained as a motivational concept and emphasizes relationships with behavioral consequences. For example, previous research shows that work engagement is positively related to organizational commitment and negatively related to intention to quit (Saks, 2006).

The women interviewed in our qualitative study discussed facing adversity and how it made them question their career choices. However, they prevailed in the profession because they found meaning and challenges, felt needed and valued, and were able to use their unique skills and capabilities. As Kahn (1990) had theorized, work provided them a sense of meaning and a sense of being worthwhile, useful and valuable. Meaningfulness is influenced by tasks, roles and work interactions. Tasks within a role that provide challenges or autonomy and have clear goals influence engagement, as do roles that carry status or influence. Work interactions that provide employees respect and a sense of worthwhileness promote engagement.

Women who persist describe finding meaning in their engineering careers, being continuously challenged, and having positive interactions with others, resulting in feelings of value and worthwhileness. Thus, engagement is hypothesized to be the process by which women persist in an engineering career despite adversity.

***Hypothesis 4: Engagement positively impacts a woman's persistence in the engineering profession***.

In the interviews with women engineers who persisted, we found no evidence that one's manager, supervisor or boss directly impacted their choice to persist in engineering. However, there was evidence that a difficult manager may push women into leaving the engineering profession. For example, a 35 year old woman who previously worked 12 years in engineering, discussed the reasons she left after having her first child:

*Her parting words (on taking maternity leave) to me were—don't do anything without talking to me first, which told me she'd find me something else, part time. Actually, that's how I read it. So I went in to maternity leave, fully thinking I was going to go back doing something part time. Then, I went in about two months into maternity leave, and she's like nope, you have to come back full time or nothing, which was really frustrating because (Company X) preaches all over the Internet about diversity… Then, we (my husband and I) decided that I would stay home, and it was actually really hard because I wasn't mentally prepared. I think I had some post-partum depression, and it was winter. It's just a major life change, and I kept thinking that that woman—that mean woman at Company X—is going to decide my future—and she did. She had decided my future by not letting me come back*.

And for a college business professor who previously worked 11 years in engineering, it seemed to be all about her bosses' impact on her:

*Well, I had some good bosses, but some very bad bosses. I think that it's so important to your career… is who you're working for. I think I had very little confidence, and I kind of waited for the other shoe to drop that somebody was going to find out that I really didn't know what was going on, and so if I had a boss who wasn't confident in me, who treated me with no respect, then I got into that completely… I needed to have somebody tell me I was good before I believed it*.

In addition to the data we collected via interviews above, a number of recent empirical studies have focused on the development of factors impacting engagement, concluding that the relationship with one's supervisor is directly related to one's level of engagement (Bakker and Bal, 2010; Rich et al., 2010). Coaching from one's supervisor and performance feedback impacts engagement (Schaufeli and Bakker, 2004) as does perceived supervisor support (Saks, 2006). Thus, we hypothesize that:

***Hypothesis 5: A positive relationship with one's supervisor positively impacts work engagement***.

In response to our question on why she stayed in the engineering profession, a technical manager with 30 years of experience in engineering describes how the ongoing assignments created a synergistic effect between her belief in herself and persisting in the profession:

*They kept giving me assignments that I thought were challenging because it's not the industry (as the reason I stayed), I'm not an auto lover. I appreciate the vehicles and whatnot, the technology, but some people are car buffs and that's why they stay. I'm not a car buff, but they were giving me assignments that were brand new. You know, Greenfield, nobody had done before. This is a whole new job, this is a whole new thing that we need to now deal with… And I think that if I believe I can do something then I can do it. And just because somebody tries to stop me, it's usually not enough*.

Because engaged employees experience positive feelings, there is a natural association with personal resources including self-efficacy (Bakker and Demerouti, 2008), where self-efficacy is the belief in one's ability to succeed (Bandura, 1977). Previous research has shown that self-efficacy is an antecedent to engagement (Christian and Slaughter, 2007). Thus, we hypothesize:

***Hypothesis 6: Self-efficacy positively impacts work engagement***.

In the interviews, it became clear that self-efficacy played a key role in persistence.

A technical manager with 30 years of experience, said the following on why she stayed:

*It has to be something built into my personality or I'm just so stubborn that I refuse to leave when they want me to leave. My father would tell you that I'm really stubborn and so I have to put that one on the list. I think the other thing is that… I happen to believe in myself even when no one else does*.

Another technical manager with 24 years of experience discusses herself and why she stayed:

*So I took an interview and they sent me for psychological screenings… One of the things the guy told me was that my personality is such that I have confidence, I'm willing to go out on a limb and do whatever… I stayed because I think it's a personality thing. My nature is to make the best of what I've got. If I have options to steer my way towards one thing or another towards something I enjoy more, as compared to just quitting and leaving and going someplace else. Also in my personality is a lack of willingness to feel like I failed at something. In a way, quitting makes me feel like I've failed. I'd much rather try to actually influence something than to just quit it*.

In our qualitative study, we found many examples of women expressing self-efficacy related to finding new work opportunities, dealing with work issues and managing the work/life interface. In contrast, women who opted out of engineering careers told stories where uncertainty, confusion, self-doubt or low confidence predominated. Thus, we hypothesize:

***Hypothesis 7: Self-efficacy positively impacts persistence***.

Findings from previous studies show that women leave professional careers at a higher rate than men and that four in 10 highly qualified women leave work voluntarily at some point in their careers, influenced by both push and pull factors (Hewlett and Luce, 2005). Push factors include the lack of job satisfaction, lack of opportunity and excessive demands, while pull factors include family pressures and personal health. Among highly qualified women off-ramped from their careers, Hewlett and Luce (2005) found 93% intending to return. Mainiero and Sullivan (2005) use the term “kaleidoscope career” to distinguish women's varied career patterns from men's more linear patterns. Women are more apt to construct careers that suit their own objectives, needs, and life criteria and more often make choices influenced by relationships and self-fulfillment (O'Neill and Bilimoria, 2005). These models are of particular relevance to both work and non-work realms and salient to this study addressing women's retention in the engineering profession as they explain career interruptions, gaps, topping out, and opting out.

The kaleidoscope career model discusses career decisions based on three parameters: authenticity, balance and challenge. As a kaleidoscope has three mirrors that create an infinite number of patterns, the kaleidoscope career model has three unique parameters reflecting an infinite number of career patterns of a woman's career (Mainiero and Sullivan, 2005). The kaleidoscope career model discusses how women's career decisions include interconnected aspects such as children, spouses, aging parents, friends, and work colleagues. Transitions occur throughout a woman's life and these transitions (such as having children) may impact career decisions and persistence in the engineering profession. Based on these insights, we hypothesize that:

***Hypothesis 8a: Age positively impacts persistence for woman in the engineering profession***.

***Hypothesis 8b: Number of children negatively impacts persistence for woman in the engineering profession***.

## Method

### Ideal self pilot study

The ideal self has been theorized as one's personal vision (Boyatzis and Akrivou, 2006) and we sought to empirically validate the theory with a measure for the ideal self. The development of the measure used the research paradigm as suggested by Churchill (1979). The initial instrument contained 32 items built on theory that were measured on a 7-point Likert scale. Doctoral students at a Midwestern university completed the initial instrument and participated in a focus group to provide feedback. Twenty items were selected for appropriateness, uniqueness, and ability to convey the concept of the ideal self (Boyatzis et al., 2010). Next, a pilot study was undertaken to assess the validity and reliability of the measure. The survey instrument included the 20 items along with demographic questions. Respondents were asked to “think about your ideal life in 10–15 years” and how it might include, “your legacy,” and “sense of purpose.” The survey was distributed to members of four non-profit organizations familiar with the first author (*n* = 96) and to business students at a Midwestern university (*n* = 16), resulting in 112 completed instruments.

Analysis of the pilot data yielded a scale with five theorized factors as shown in Table 1 and detailed in the Supplementary Material. The ideal self-hope factor includes eight items relating to one's feelings of possibilities; the ideal self-sense of purpose scale includes four items assessing relative priorities related to one's legacy or calling; the ideal self-holistic vision assesses family and relationships using four items; the ideal self-deeper meaning with two items relates to one's values; and the ideal self-fun includes two items relating to the importance of fun in leisure.

**Table 1:** **Pilot study measurement model and correlations for the ideal self as a 5-factor scale^a^**.

	**Measure**	**CR**	**AVE**	**1**	**2**	**3**	**4**	**5**
1	Ideal self-hope	0.897	0.526	**0.832**				
2	Ideal self-sense of purpose	0.861	0.608	0.445	**0.758**			
3	Ideal self-holistic vision	0.847	0.581	0.270	0.527	**0.758**		
4	Ideal self-deeper meaning	0.822	0.698	0.458	0.362	0.371	**0.654**	
5	Ideal self-fun	0.852	0.743	0.369	0.561	0.561	0.351	**0.565**

* *n = 112. Reliability coefficients are reported along the diagonal*.

### Participants and procedures

A survey was developed, based on the hypothesized model in Figure 1, including the 5-factor ideal self-scale (Boyatzis et al., 2010). Data was collected from professionals with engineering degrees by sending an email with the survey link to approximately 20 executives, managers, and engineers who were past work colleagues, schoolmates and/or friends of the first author. The email asked them to take the survey and to forward the survey to anyone they knew who had an engineering degree. Included in the original emails, were executives in four Fortune 500 companies that employ a considerable number of engineers. Following receipt of the email, several professional groups sent the link to their members and/or included the survey link in e-newsletters. These included several sections of the Society of Women Engineers, Phi Sigma Rho (an engineering sorority), and the IEEE (Institute of Electrical and Electronic Engineers) Women in Engineering Network, resulting in 495 surveys used in the analysis.

### Measures

Persistence in an engineering career was measured several ways within the survey. First, the respondents were asked if they had ever chosen to leave an engineering career, with responses simply “yes” or “no.” Next, we asked about years employed as an engineer and what current position was held with choices of engineering, engineering manager, higher level position normally afforded one based on a successful engineering career, and other options related to leaving the engineering profession including returned to school full time, stay-at-home mother etc. For use within the structural equation model (SEM), the construct career commitment (Blau, 1999) was adapted for the engineering profession as a representation for career persistence. As described by Blau, a profession is a type of occupation where characteristics such as expertise, autonomy and regulation of its member transcend employing organizations which describes engineering. Career commitment was originally developed by Blau (1985) as a differentiating construct from organizational commitment and for this reason, suits the current study on women persisting in the engineering profession, not necessarily in a specific job or a specific organization. Reliabilities for the scale have been reported at 0.82 and greater and have been examined for age, tenure in career, and marital status (Blau, 1988, 1999; Goulet and Singh, 2002; Duffy et al., 2011). The four items used in the present study were: (a) “I definitely want a career for myself in engineering or technical management”; (b) “If I could do it all over again, I would choose to work in engineering; (c) “I would recommend a career in engineering to others”; (d) “I am not disappointed that I ever entered the engineering profession.” Items were scored on a 5-point scale 1 = I disagree a lot to 5 = I agree a lot.

Each of the five ideal self-scales were included in the survey, using a 7-point scale where 1 = Strongly Disagree and 7 = Strongly Agree. These are detailed in the Supplementary Material. For example, the ideal self-hope scale included eight items such as: “I feel inspired by my vision of the future”; and “My vision reflects many possibilities.”

For engagement, the Utrecht Work Engagement Scale (UWES) work and well-being survey (Schaufeli et al., 2006) was adapted using 15 items such as (a) “At my work, I feel bursting with energy”; (b) “I find the work that I do full of meaning and purpose”; (c) “Time flies when I am working”; (d) “To me, my job is challenging” and (e) “I get carried away when I am working.” Items were scored on a 7 point basis with 1 = Never, 2 = Almost Never (A few times a year or less), 3 = rarely (Once a month or less), 4 = Sometimes (a few times a month), 5 = Often (Once a week), 6 = Very Often (A few times a week), and 7 = Always (everyday).

The Schwarzer and Jerusalem (1995) general self-efficacy scale was used as it is designed to assess self beliefs related to coping with difficult demands in life. For women in the engineering profession, these difficult demands include, but are not limited to difficult work situations and work-life issues (Frehill, 2008; Hewlett et al., 2008; Fouad and Singh, 2011; Buse et al., 2013). The scale was developed to explicitly refer to personal agency and has been used in thousands of studies showing discriminant and convergent validity. Specific items used included: (a) “I am confident that I could deal efficiently with unexpected events”; (b) “Thanks to my resourcefulness, I know how to handle unforeseen situations”; (c) “If I am in trouble, I can usually think of a solution”; (d) “I can usually handle whatever comes my way.” Items were scored on a 4-point basis with 1 = Not at all True, 2 = Hardly True, 3 = Moderately True and 4 = Exactly True.

For optimism, we used the LOT-R or life orientation test revised (Scheier et al., 1994) adapted to five items: (a) “If something can go right for me, it will”; (b) “I'm always optimistic about my future”; (c) “I usually expect things to go my way”; (d) “I usually count on good things happening to me”; and (e) “Overall, I expect more good things to happen to me than bad.” Scoring for the LOT-R was on a 5-Point scaled with 1 = I agree a lot to 5 = I disagree a lot.

The hope state scale developed by Snyder et al. (1996), was used in this study. The six-item scale included (a) “If I should find myself in a jam, I could think of many ways to get out of it”; (b) “At the present time, I am energetically pursuing my goals”; (c) “If I should find myself in a jam, I could think of many ways to get out of it”; (d) “At the present time, I am energetically pursuing my goals”; (e) “There are lots of ways around any problem that I am facing now”; (f) “Right now I see myself as being pretty successful”; (g) “I can think of many ways to reach my current goals”; and (h) “At this time, I am meeting the goals that I have set for myself.” An 8-point scale was used for scoring where 1 = Definitely False 2 = Mostly False 3 = Somewhat False 4 = Slightly False 5 = Slightly True 6 = Somewhat True 7 = Mostly True, and 8 = Definitely True.

As defined by Cheek et al. (2002), core identity is an unconscious set of enduring individual characteristics. Identity was measured using three items: (a) “Knowing that I continue to be essentially the same inside even though life changes”; (b) “My self-knowledge, my ideas about what kind of person I really am”; and (c) “My personal self-evaluation, the private opinion I have of myself.” The 5-point scale used for scoring was: 1 = Not important to who I am 2 = Slightly important to my sense of who I am 3 = Somewhat important to my sense of who I am 4 = Very important to my sense of who I am and 5 = Extremely Important to my sense of who I am.

Leader–Member Exchange was chosen to measure a woman's perceived relation with her manager (Graen and Uhl-Bien, 1995). Seven items were included such as (a) “Do you know where you stand with your leader… do you usually know how satisfied your leader is with what you do?” with scoring as 1 = Rarely 2 = Occasionally 3 = Sometimes 4 = Fairly Often 5 = Very Often; and (b) “How well does your leader understand your job problems and needs?” with scoring as 1 = Not a bit 2 = A Little 3 = A Fair Amount 4 = Quite a Bit 5 = A Great Deal.

### Data analysis

Exploratory factor analysis (EFA) and confirmatory factor analysis (CFA) were employed to verify the uni-dimensionality, the validity, and the reliability of the model constructs. SPSS for Windows (PASW Statistics Gradpack 17.0, 2009) was used to conduct the EFA on the measures using principal axis factoring and Promax oblique rotation method. CFA and the SEM were completed using AMOS. Mediation was tested using the criteria established by Preacher and Hayes (2008).

## Results

### Descriptive statistics

The means, standard deviations, reliabilities and correlation between the study variables are shown in Table 2. All variables have acceptable reliabilities including two of the newly developed ideal self-scales which exceed the 0.60 exploratory criteria (Hair et al., 2010).^1^

**Table 2:** **Means, standard deviations, cronbach's alphas and correlations for the model variables^b^**.

		**Mean**	***SD***	**1**	**2**	**3**	**4**	**5**	**6**	**7**	**8**	**9**
1	Self-efficacy	3.45	0.44	**0.81**								
2	Identity	4.09	0.64	0.20	**0.67**							
3	Optimism	3.81	0.78	0.33	0.15	**0.87**						
4	Hope	6.59	0.86	0.56	0.18	0.48	**0.87**					
5	Ideal self-hope	5.99	0.81	0.28	0.16	0.37	0.49	**0.91**				
6	Ideal self-sense of purpose	5.37	1.19	0.28	0.24	0.31	0.43	0.59	**0.79**			
7	Leader–member exchange	3.60	0.84	0.17	0.02	0.20	0.26	0.17	0.06	**0.92**		
8	Career commitment to engineering	4.03	0.89	0.23	0.14	0.21	0.34	0.26	0.17	0.22	**0.80**	
9	Engagement	5.47	1.14	0.29	0.14	0.31	0.47	0.38	0.36	0.36	0.37	**0.96**

b *n = 495. Reliability coefficients are reported along the diagonal*.

The confirmatory factor analysis showed that the model had acceptable fit with *n* = 495 where χ ^2^ = 2844, *df* = 1230, χ ^2^/*df* = 2.32, CFI = 0.904, RMSEA = 0.052, PCLOSE = 0.152. Convergent and discriminant validity were established using the criteria from Hair et al. (2010).

### Survey respondents

The sample of 495 women with engineering degrees was further analyzed to understand differences related to age, employment status, marital status, and number of children. Table 3 summarizes the employment of the 495 women by age. Forty-six percent of the respondents had described their current employment an engineer. Twenty-five percent described themselves as technical managers and 14% were executives. Sixteen percent described their current role as not an engineer, nor in any position related to engineering, with 37 women or 7.4% identifying their current job as non-engineering, five were unemployed and looking, one was unemployed and not looking, five self-described themselves as stay-at-home moms, one was retired, one was a student in a non-engineering degree program, 14 were in school seeking another engineering degree, and 13 listed “other.”

**Table 3:** **Employment statistics by age for women with engineering degrees**.

**Sample**			**Current employment**				**Years worked in engineering**							
**Age range**	**N**	**%**	**Engineer**	**Manager^a^**	**Executive^b^**	**Other^c^**	**Never**	**1–5**	**6–10**	**11–15**	**16–20**	**21–25**	**26–30**	**30+**
21–25	84	17%	54	6	3	21	7	77	0	0	0	0	0	0
26–30	96	19%	61	11	9	15	5	63	28	0	0	0	0	0
31–35	65	13%	33	14	11	7	0	8	34	23	0	0	0	0
36–40	61	12%	25	20	10	6	1	2	9	34	0	0	0	0
41–45	76	15%	22	29	12	13	2	2	9	9	26	28	0	0
46–50	63	13%	23	23	13	4	2	0	3	6	10	28	14	0
51–55	44	9%	7	18	9	10	0	1	2	4	1	6	15	15
56–60	5	1%	0	4	0	1	0	0	0	0	2	1	2	0
61+	1	0.20%	1	0	0	0	0	0	0	0	0	0	0	1
All	495	100	226	125	67	77	17	153	85	76	39	63	31	16
			46%	25%	14%	16%	3%	31%	17%	15%	8%	13%	6%	3%

a “I am currently employed in a technical management or engineering management role.”

b “I am currently employed in a position that was a normal promotional move from my engineering career (but not in engineering or technical management).”

c *Not an engineer or any position related to engineering or unemployed or a student*.

It is important to note that more than 49% of the women were 35 years of age or younger.

Numerous engineering degrees were identified including chemical engineering (46%), mechanical engineering (12%), electrical engineering (8%), civil engineering (5%), industrial (4%), and biomedical engineering (4%). Table 4 summarizes the demographic data related to age, marital status and number of children. In total 25% never married, 18% were married with no children, 45% were married with children, 6% divorced or separated, and 5% living with a partner. Forty seven pwercent of the women engineers had no children, 11% had one child, 29% had 2 children, 10% had 3 children, and 2% had four or more children. Women 30 years of age and younger comprised 36% of the participants. Only 4% of these women (30 years of age and younger) had children while 77% of women ages 31–50 years of age had children. Women ages 31–50 years of age comprised 53% of the participants with women 51 and older comprising 10% of the participants and 75% of them reported having children.

**Table 4:** **Marital status and number of children for women with engineering degrees**.

**Sample**			**Marital status**				**Children**				
**Age range**	**N**	**%**	**Single**	**Married**	**Divorced**	**Living with partner**	**0**	**1**	**2**	**3**	**4+**
21–25	84	17%	63	14	1	6	81	2	1	0	0
26–30	96	19%	37	52	0	7	80	10	5	1	0
31–35	65	13%	14	47	2	2	28	9	22	6	0
36–40	61	12%	4	49	7	1	8	8	33	10	2
41–45	76	15%	4	64	6	2	17	11	33	11	4
46–50	63	13%	5	50	4	4	9	8	35	10	1
51–55	44	9%	2	36	4	2	11	6	13	11	3
56–60	5	1%	0	2	3	0	0	0	2	2	1
61+	1	0.2%	0	0	1	0	0	0	1	0	0
All	495	100	129^a^	314	28	24	234	54	145	51	11
			26%	63%	5.7%	4.8%	47.3%	10.9%	29.3%	10.3%	2.2%

a *Includes 3 widows*.

### Analysis for common method bias

Common method bias (CMB) is a concern when conducting self-reported research as it refers to variance that is attributable to the measurement method rather than to the constructs. Most researchers agree that common method variance is a potential problem in behavioral research (Podsakoff et al., 2003). To determine if the common method for data collection impacts the measurement model, Pavlou et al. (2007) discuss examination of the correlation table of the latent variables and CMB may be present if correlations are above 0.90. As shown in Table 2, the correlations of the study variables are all below this 0.90 standard. Further to assess for methods bias a confirmatory factor analysis was conducted in which the baseline model included a CMB factor where each item was converted to a single item construct (Podsakoff et al., 2003). Each of these single item constructs is linked to a common method factor (CMF). The variance associated with the measurement model was more than 2.5 greater than the variance associated with the CMF and it is concluded that common method variance does not bias the results of this study.

### Direct effects within the structural equation model

**Figure 2:**
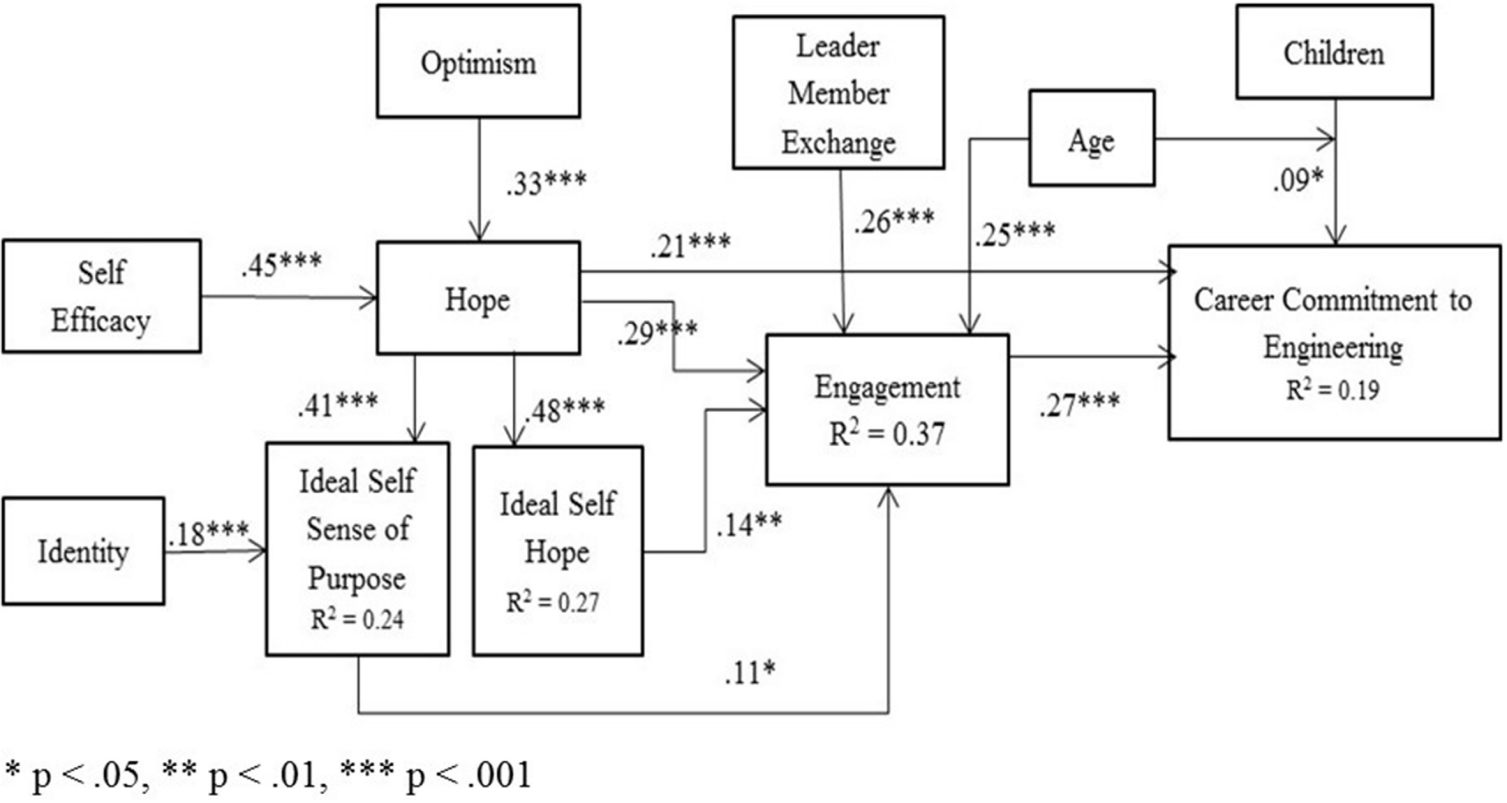
**Standardized solution for women's career commitment to engineering**.

Figure 2 shows that hypotheses 2a, 2b, 3a, 3b, 4, and 5 are supported as there are direct significant effects within the SEM. Career commitment to engineering is positively impacted by engagement with β = 0.27, *p* < 0.001 (H1) and hope β = 0.21, *p* < 0.001. Engagement is significantly impacted by leader–member exchange β = 0.26, *p* < 0.001(H2), age β = 0.20, *p* < 0.001, hope β = 0.28, *p* < 0.001, the ideal self-hope β = 0.14, *p* < 0.01 and the ideal self-sense of purpose β = 0.11, *p* < 0.05. The ideal self-sense of purpose is positively impacted by hope β = 0.36, p < 0.001 (H5a) and identity β = 0.15, *p* < 0.001(H5b). Hope is positively impacted by self-efficacy β = 45, *p* < 0.001(H6a) and optimism β = 0.33, *p* < 0.001(H6b).

No support was found hypotheses 8a or 8b as career commitment is not directly or indirectly impacted by either age or number of children. There was an interaction effect of age and children as described in Figure 2.

### Mediation testing

Hypotheses 6, and 7 are supported by mediation testing using the method suggested by Preacher and Hayes (2008). Engagement fully mediates the following relationships to career commitment: leader–member exchange, self-efficacy (H7) and optimism. Engagement partially mediates the impact of hope on career commitment. Additionally, we found that hope fully mediates the impact of self-efficacy and optimism on engagement. H1 is not supported; instead, we find that the ideal self-hope and the ideal self-sense of purpose directly impact engagement which directly impacts career commitment. Table 5 includes the direct, indirect and total effects of leader–member exchange, self-efficacy, hope and the two ideal self-constructs on engagement and career commitment to engineering.

**Table 5:** **Direct, indirect and total effects of variables on work engagement and career commitment to engineering**.

	**Engagement**			**Career commitment to engineering**		
	**Direct**	**Indirect**	**Total**	**Direct**	**Indirect**	**Total**
Self-efficacy		0.18^**^	0.18^**^		0.14^**^	0.14^**^
Optimism		0.13^**^	0.13^**^		0.10^*^	0.10^*^
Hope	0.28^***^	0.12^**^	0.40^***^	0.21^***^	0.10^*^	0.31^***^
Ideal self-sense of purpose	0.10^**^		0.10^**^		0.03	0.03
Ideal self-hope	0.14^**^		0.14^**^		0.04	0.04
Leader–member exchange	0.26^***^		0.26^***^		0.07^*^	0.07^*^

* *p < 0.05*,

** *p < 0.01*,

*** *p < 0.001*.

### Interaction effect

**Figure 3:**
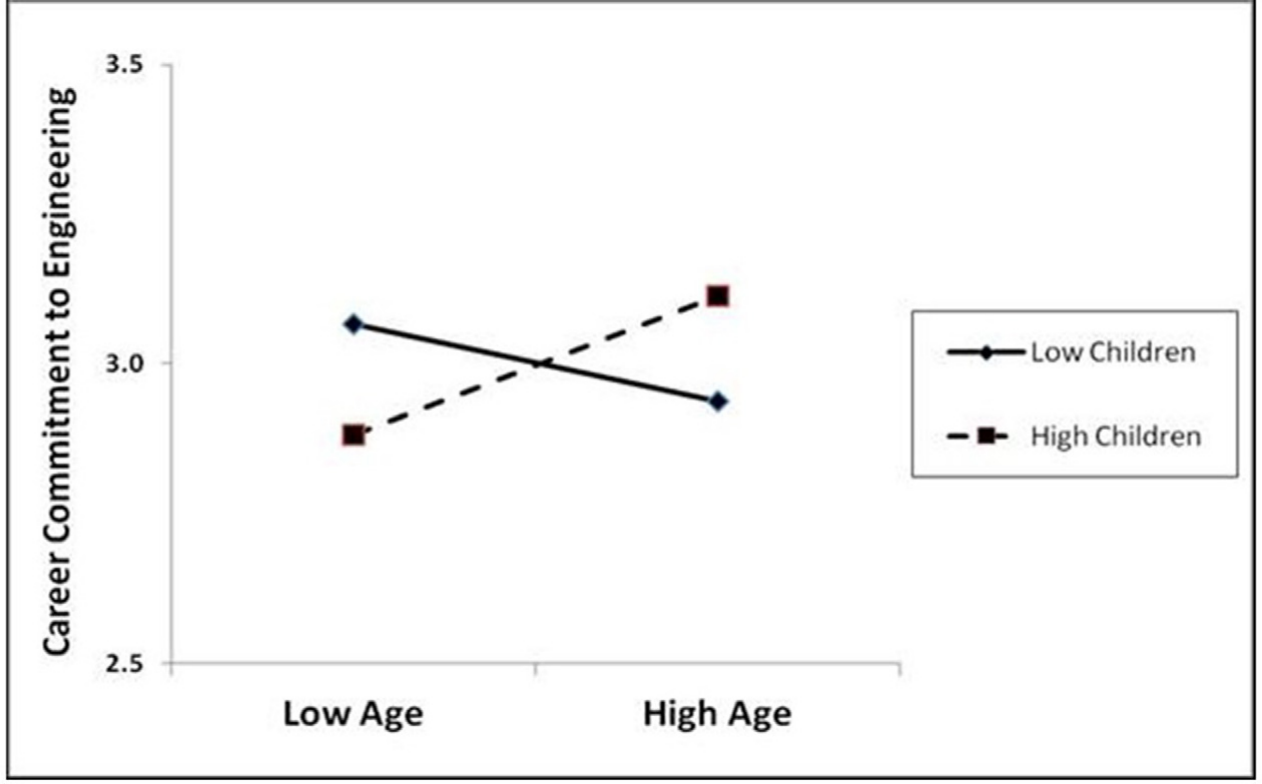
**Interaction effects of age and children on career commitment to engineering**.

Since no support was found for age and number of children directly impacting career commitment (H8a and H8b), we tested for an interaction effect. The interaction of age and number of children was found to influence career commitment as shown in Figure 3. For those women with fewer children, career commitment to engineering decreases with age. For women with more children, career commitment to engineering increases with age.

### Model fit

The model, as shown in Figure 2, is found to have acceptable fit (Hu and Bentler, 1999; MacCallum and Austin, 2000; Hair et al., 2010) with χ ^2^ = 46.6, *p* = 0.004, *df* = 24, χ ^2^/*df* = 1. 942, RMSEA = 0.044, pclose = 0.690 and CFI = 0.984.

## Discussion

This study contributes three important findings to the literature on personal vision, work engagement and women's careers. First, the study empirically validates that a personal vision, as operationalized as the ideal self, is comprised of self-efficacy, optimism, hope and core identity and that the ideal self directly impacts work engagement. Second, the study shows that work engagement is the mediating mechanism linking career commitment to self-efficacy, hope, and leader–member exchange. Lastly, we find that a woman's career commitment is influenced not only by work engagement, but is impacted by her relationship with her manager and an interaction effect between her age and number of children.

A key contribution of the study is that these findings support theoretical development that the ideal self is comprised of identity and hope, with self-efficacy and optimism components of hope (Boyatzis and Akrivou, 2006). As theorized by Boyatzis (2008), change occurs when one acknowledges discrepancies between one's ideal self and one's real self. The acknowledgement often results from a tipping point, where women discover that their real self is not aligned with their ideal self. This discovery motivates them to leave engineering careers. For the women who persisted, they described themselves in engineering terms and discussed their work in engineering as challenging and meaningful. Their ideal self was aligned with their real self, here conceptualized as work engagement. The ideal self directly impacts work engagement and greater work engagement results in greater commitment to engineering.

The present study also contributes to understanding how work engagement is a mediating mechanism related to women's persistence in engineering. Engagement in a work role has become popular in practice as engagement has been related to organizational benefits (Saks, 2006). Here, we add empirical evidence to recent work that views engagement as a motivational concept with behavioral consequences (Rich et al., 2010), as women who are engaged in their engineering work are more likely to persist in their engineering career. The retention of these women in the engineering profession not only benefits organizations, but benefits women and society as a whole (Margolis et al., 1999/2000). Women benefit economically as the number of engineering jobs will continue to grow and salaries are relatively high (Bureau of Labor Statistics, 2014b). Organizations and society benefit from the broadened perspective and diversified talent women bring to the field (Margolis et al., 1999/2000).

The results of this study not only validate prior theory on factors impacting engagement but add to theory, as age, hope, optimism and the ideal self are shown to be impact work engagement. Self-efficacy and the relationship with one's manager also impact engagement, supporting earlier theoretical development and empirical studies (Saks, 2006; Bakker and Demerouti, 2008; Rich et al., 2010).

Our study extends previous research on women's careers by addressing why women persist as opposed to identifying the reasons women leave professional roles (Hewlett and Luce, 2005; Frehill, 2008; Hewlett et al., 2008; Fouad and Singh, 2011). Most importantly, the findings from the present research study show that the reasons women persist are not the inverse of the reasons women leave. Previous research shows that women leave the engineering profession in large measure because of difficult work conditions and environments (Frehill, 2008; Hewlett et al., 2008) and specifically due to the lack of supportive organizational practices (Singh et al., 2013). Yet, women who persist in engineering face the same challenging work conditions and environments but overcome these challenges (Buse et al., 2013). The women who persist do so because they are engaged in their work. The challenges, novelty and meaningfulness they find in their work seem to allow them to overcome difficult workplaces.

While the kaleidoscope career model (Mainiero and Sullivan, 2005) suggests that women trade challenge for balance, this study suggests that if women are appropriately challenged and find meaning and engagement in their work, they will find the appropriate balance.

The findings related to marital status and number of children may surprise those who theorize that women leave the workforce for marriage and children. Most women in the study's sample were married (63%), and for women over 30 years of age, 79% were married. More than three quarters of the women over 30 years of age had at least one child and 63% had two or more children. Further, the number of children had no direct impact on either engagement nor on career commitment, but an interaction effect occurred such that women with more children increased their commitment to engineering as they aged. In contrast, only age had an impact on engagement with older women being more engaged in their work, supporting the earlier discussion that women who persist do so because they find meaning and challenge in their work. The differences in the responses related to age are important in this study and augment theoretical development related to women's career patterns.

### Practical implications

As mentioned at the beginning of this paper, corporations including Google are seeking a more diverse workforce. Based on this study, we offer three broad practical suggestions to improve recruitment, retention and advancement of women in engineering and other STEM professions. First, these findings emphasize the importance of professional and leadership development for women in STEM professions. Next, the study emphasizes how important work engagement is for STEM women to persist in their profession. Lastly, we emphasize the need for supporting relationships in organizations that include STEM workers.

Our first suggestion is that women in the STEM professions have the opportunity to create a personal development plan. As found in our study, women who could articulate a personal vision were more likely to be engaged in their work and committed to the profession. Formalizing the development plan through a process aids in understanding of her role and her future work.

Ely et al. (2011) recommend development programs designed specifically for women in leadership. These types of programs enable women to understand how their careers are impacted by second generation gender bias. We urge organizations to provide professional and leadership development opportunities for women in STEM occupations. These programs should be developed to provide women with the opportunity to develop a personal vision and to help them understand how bias impacts them in the STEM professions. Additionally, skills and competencies important to achieving their vision can be developed, including self-efficacy. These development programs are likely to give organizations a differential advantage when recruiting women to their organizations. The programs will enable STEM women working in their organizations to recognize and overcome barriers to achievement. Hope is a powerful motivating emotion. As we found, women who are hopeful are more engaged at work and committed to the STEM professions.

In our interviews with women who persisted in the engineering profession, we heard stories of how the work provided ongoing challenges, novelty and the opportunity for continuous learning. Higher levels of work engagement lead to higher levels of commitment to the engineering profession. For corporations seeking to recruit and retain women engineers, the work should be emphasized and matched to the women's background and interests. Our own experience is that women are often placed into roles such as quality engineering, which requires less technical skills and no direct line to advancement.

Supporting relationships are needed in any workplace. The under-representation of women in engineering, with only 1 in 10 engineers being women, creates an environment where women are the minority and often do not get the support they need from their managers or their colleagues—as we found in our study. Organizations seeking to retain and advance women engineers should provide mentors and sponsors to aid women in the workplace.

Our study shows that women who have support from their leaders are more engaged at work and more likely to persist. Our own experience in industry has been that people who are successful in technical roles are the ones promoted to leadership roles. Skills needed to be good at technology are not the same skills that allow one to be a good leader. We recommend that all leaders in organizations be provided with leadership development to ensure that women (and men) are supported in the workplace.

This study aligns with the work of other researchers who suggest organizational changes are necessary to retain women in the STEM professions (National Research Council, 2007; Bilimoria et al., 2008; Hewlett et al., 2008; Fouad and Singh, 2011; Bilimoria and Liang, 2012). Organizations should recognize the compelling business case associated with increasing gender diversity at all levels of the organizations including the number of women in senior leadership (Catalyst, 2004; Ernst and Young, 2009; McKinsey and Company, 2010). This study shows that organizations wanting to recruit, retain and advance STEM women should provide women with the opportunity for personal development, ensure that the work is challenging and novel, and that women are matched with managers and mentors who provide support.

### Directions for future research

Future studies are recommended to explore how one's personal vision impacts career and life choices. The ideal self-scale can be used to empirically validate relationships between a personal vision and these choices. Studies are also recommended that can explore additional antecedents for the ideal self. Continued theory development related to one's ideal self and how one creates and maintains a personal vision is recommended with empirical studies to support the theory.

Researchers are encouraged to continue to develop theories and empirical support for factors impacting women's persistence in professional careers including engineering. Particularly, factors related to engagement in the workplace and contextual factors that aid organizations in retaining women should be explored, including additional individual factors as well as institutional, social and cultural factors (National Research Council, 2007). This work is especially recommended for women engineers as well as women in other STEM professions as it would build upon the current findings and support practical solutions to the enduring problem of women's under-representation in these professions. Research is also recommended comparing the current findings on women in the engineering profession to their male peers. Finally, additional research is recommended on how self-efficacy and self-confidence are developed within professional roles, and how educational programs at the undergraduate and graduate levels as well as workplaces can facilitate their development.

### Conflict of interest statement

The authors declare that the research was conducted in the absence of any commercial or financial relationships that could be construed as a potential conflict of interest.

## Footnotes

^1^Descriptive statistics are included in Table 2 for the constructs that have significant effects in the structural equation model (Figure 2). The ideal self-hope and the ideal self-sense of purpose are shown because they significantly impacted engagement. Since the other three ideal self-scales did not have any impact they are not included in Table 2.
