# Supplementary material for: Personal vision: enhancing work engagement and the retention of women in the engineering profession
Source: Front Psychol. 2014 Dec 8;5:1400. doi: 10.3389/fpsyg.2014.01400 (PMC4259005; doi:10.3389/fpsyg.2014.01400)
Supplement: Supplementary file 1 [file DataSheet1.DOCX]

# Appendix

Ideal Self (IS) Scale - Boyatzis, Buse, & Taylor 2010

Using the scale 1 = strongly disagree, 7= strongly agree

IS Hope – this construct includes items that focus on the possibilities and the feelings of one’s vision

I feel inspired by my vision of the future.

My vision reflects many possibilities.

My vision includes my work in terms of my jobs and career.

I am excited about my vision.

I feel hopeful about my vision.

I feel optimistic about my vision.

I have a clear vision of my desired future.

I see many possibilities in my future

IS Sense of Purpose - this construct includes items that focus on how well one’s personal vision includes one’s understanding of a purposeful life.

My vision includes relative priorities of things important to me.

My vision includes my desired legacy in life.

My vision of the future reflects the things most important to me.

My passion, calling, and sense of purpose are clear to me.

IS Holistic Vision – this construct include items that frame a complete personal vision.

My vision includes my values and philosophy.

My vision includes my contributions to others and the community.

IS Deeper Meaning – this construct includes four items related to health and relationships.

My vision includes my family relationships.

My vision includes my physical health.

My vision includes my intimate/love relationships.

My vision includes my spiritual health.

IS Fun – this construct includes two items that constitute the playful part of a personal vision.

My vision includes fun activities.

My vision includes leisurely activities.
